# Red Blood Cell Distribution Width Is Associated with Severity of Leukoaraiosis

**DOI:** 10.1371/journal.pone.0150308

**Published:** 2016-02-26

**Authors:** Han-Bin Lee, Jinkwon Kim, Seung-Hun Oh, Sang-Heum Kim, Hyun-Sook Kim, Won-Chan Kim, Soonhag Kim, Ok-Joon Kim

**Affiliations:** 1 Department of Neurology, CHA Bundang Medical Center, CHA University, Seongnam, South Korea; 2 Department of Radiology, CHA Bundang Medical Center, CHA University, Seongnam, South Korea; 3 Institute for Bio-Medical Convergence, College of Medicine, Catholic Kwandong University, Gangneung-si, Republic of Korea; 4 Catholic Kwandong University International St. Mary’s Hospital, Incheon Metropolitan City, Republic of Korea; Instituto de Ciências Biomédicas / Universidade de São Paulo - USP, BRAZIL

## Abstract

Red blood cell distribution width (RDW) is one of the routine hematologic parameters reported in the complete blood count test, which has been recognized as strong prognostic marker for various medical conditions, especially cardiovascular disease. We evaluated that RDW was also associated with the leukoaraiosis; common radiological finding of brain and that has been strongly associated with risk of stroke and dementia. In the present study, we included 1006 non-stroke individuals who underwent brain MRI and routine complete blood count test including RDW. Fazekas scale was used to measure the severity of leukoaraiosis based on fluid-attenuated inversion recovery image, and the severity was dichotomized to mild-degree (Fazekas scale: 0–1) and severe-degree leukoaraiosis (Fazekas scale: 2–3). Univariate and multivariate logistic regression models were constructed to evaluate independent risk factor for severe-degree of leukoaraiosis. Mean age of 1006 subjects was 64.34 ± 9.11 year, and mean of RDW was 12.97 ± 0.86%. The severe-degree of leukoaraiosis (Fazekas scale ≥ 2) was found in 28.83%. In the multivariate logistic regression, 4^th^ quartile of RDW (> 13.3%) were significantly associated with the presence of severe-degree of leukoaraiosis (adjusted odds ratio, 1.87; 95% confidence interval, 1.20–2.92) compared to the 1^st^ quartile of RDW (< 12.5%). The significance was not changed after adjustments for hemoglobin and other hematologic indices. These findings suggest that RDW is independently associated with severity of leukoaraiosis.

## Introduction

The red blood cell distribution width (RDW) is one component of the complete blood cell count, which is easily measured from a peripheral blood sample. The RDW represents the variance in the volume of circulating red blood cells (RBCs). An elevated RDW indicates abnormal variation in RBC size, termed anisocytosis. Anisocytosis usually results from increased or ineffective production of RBCs and excessive fragmentation or destruction of RBCs [1,2]. Therefore, RDW is primarily used to evaluate the etiology of anemia and associated hematologic conditions. However, it is recognized that RDW also has a strong prognostic value for a variety of diseases, especially acute vascular diseases [3–5]. Patients with a higher RDW, even if within the normal reference range, are at increased risk for coronary artery disease, stroke, peripheral artery disease, and all-cause death [2,6,7].

Leukoaraiosis, also known as cerebral white matter hyperintensities, is a common finding in brain imaging in the elderly, especially in those with cerebrovascular and neurodegenerative diseases [8]. Even though leukoaraiosis is non-specific radiological diagnosis, its presence corresponds well to the pathological conditions of neuronal loss, ischemic demyelination, and gliosis in brain tissue [9]. The underlying pathomechanism of leukoaraiosis is hypothesized to be diffuse small vessel disease or chronic ischemic injury of brain [8]. The clinical importance of leukoaraiosis has increasingly been recognized in recent times because of its high prevalence and strong prognostic value in several cerebral diseases [10,11]. Leukoaraiosis is considered an indicator of impaired brain function and reduced brain recovery potential following injury [12]. Individuals with leukoaraiosis are at increased risk for dementia, cognitive decline, functional disability, stroke and mortality [10]. Furthermore, recent evidence suggests that leukoaraiosis plays a causal role in the development and progression of neurodegenerative and cerebrovascular diseases [11,13].

From reviewing the literature, we find that individuals with elevated RDW and leukoaraiosis share similar risk factors and related medical conditions. Both RDW and leukoaraiosis are increased in ageing, the presence of chronic illness, inflammation and atherosclerotic diseases [3]. Endothelial dysfunction and haemostatic activation are frequently found in those people with elevated RDW or leukoaraiosis [14,15]. Recent studies have demonstrated that the RDW might be useful in evaluating the risk of dementia, a common clinical presentation of severe leukoaraiosis [16]. Based on these findings, we hypothesized that RDW may be associated with the severity of leukoaraiosis. In this study, we evaluated such associations by measurement of RDW and magnetic resonance image (MRI)-defined leukoaraiosis in non-stroke individuals.

## Materials and Methods

### Study design

This study was designed as a retrospective analysis of subjects with aged ≥ 45 years, who visited the outpatient clinic of the Department of Neurology at CHA Bundang Medical Center for routine health examination between June 2003 and August 2014. Subjects presented for medical attention because they had underlying cardiovascular risk factors, or a family history of stroke. We included only non-stroke individuals who completed brain MRI investigations, including T2-weighted and fluid attenuation inversion recovery (FLAIR) images and magnetic resonance angiography (MRA). We reviewed the medical records of all subjects in regard to demographical characteristics, laboratory results and radiological findings. Of 1372 study subjects, we excluded 366 subjects for the following reasons: (1) inadequate clinical information (n = 93); (2) no laboratory tests (n = 127); (3) no brain MRI or MRA data (n = 82); (4) previous history of stroke (n = 37); (5) abnormal neurological findings at the time of examination (n = 27). Therefore, a total of 1006 non-stroke subjects were included for analysis. Among them, the clinical and radiological information of 534 subjects were previously described elswhere [17]. The medical record or patient’s information was de-identified prior to analysis, and the Institutional Review Board (IRB) of CHA Bundang Medical Center approved the study (IRB no.: BD-2010-083).

### Collection of clinical characteristics

Data were collected on subjects’ gender, age, the presence of hypertension, diabetes mellitus (DM), current smoking, hypercholesterolemia, and coronary artery disease (CAD).

Hypertension was diagnosed when a patient was on anti-hypertensive medication following diagnosis, or had systolic blood pressure ≥ 140 mmHg or diastolic blood pressure ≥ 90 mmHg on repeated measurements. DM was diagnosed if the patient had a fasting plasma glucose ≥ 7.0 mmol/l or was being treated with anti-diabetic medications. Hypercholesterolemia was diagnosed if the patient had a total cholesterol ≥ 6.2 mmol/l, or the patient was treated with lipid lowering agents following diagnosis of hypercholesterolemia. Current smoking was defined as having smoked a cigarette within 1 year. CAD was identified when a patient had a history of acute myocardial infarction, unstable angina, angiographically confirmed coronary artery occlusive disease, coronary artery bypass graft or percutaneous coronary artery stent/angioplasty.

### Measurement of RDW and laboratory findings

RDW measurement was performed with peripheral venous blood using an automated complete blood cell counter (UniCel DxH 800, Beckman Coulter Inc., Miami, FL, USA), which simultaneously provides values for hemoglobin, MCV, mean corpuscular hemoglobin (MCH), mean corpuscular hemoglobin concentration (MCHC), platelet count, and total white blood cell count. Other collected laboratory data are total cholesterol, triglyceride, glucose, and estimated glomerular filtration rate (eGFR). The eGFR was calculated using the abbreviated Modification of Diet in Renal Disease Study equation [186 x Serum Creatinine^-1.154^ x Age^-0.203^ x (0.742 if female)] [18].

### Image analysis including measurement of leukoaraiosis

Brain MRI and MRA were performed on all study subjects using one of three 1.5-T systems (Sonata, Siemens Healthcare (n = 898); Signa Excite, GE Healthcare (n = 47); Signa HDx, GE Healthcare (n = 61)) at subjects’ own expense. To evaluate the severity of leukoaraiosis, one neurologist (HBL) and one neuroradiologist (SHK) who had experience in assessing the visual rating score of leukoaraiosis on conventional MRI [17] blindly scored the image with Fazekas’s grading scale of periventricular and subcortical white matter (0 indicates absent and 3 indicates severe leukoaraiosis) in axial FLAIR imaging (repetition time (TR)/echo time (TE): 9000/105 ms, inversion time: 2500 ms, slice thickness: 5 mm) [12,19]. The inter-rater agreement of Fazekas score was adequate (weighted Kappa: 0.754; 95% confidence interval: 0.725 to 0.784). When the two raters disagreed on the score, decision of the final score was made by further discussion between them. The presence of cerebral atherosclerosis was diagnosed based on MRA when either significant stenosis (≥ 50%) or occlusion was present in intracranial or extracranial cerebral arteries (anterior cerebral, middle cerebral, posterior cerebral, internal carotid, vertebral, and basilar arteries). The degree of stenosis in the extracranial cerebral artery was measured using the method used in the North American Symptomatic Carotid Endarterectomy Trial [20], and that in the intracranial cerebral artery was measured using the method in the Warfarin vs. Aspirin for Symptomatic Intracranial Disease study [21]. If one artery had multiple stenotic lesions, the most severe degree was selected. Silent brain infarct (SBI) was defined as a cavitated lesion in areas supplied by deep perforating arteries showing high intensity on the T2-weighted image (TR/TE: 3700/90 ms; slice thickness: 5 mm) and low signal intensity with a hyperintense rim on FLAIR image [22].

### Statistical analysis

Categorical data were expressed as number (%), and continuous data were expressed as mean ± standard deviation (SD) or median [interquartile range]. To evaluate the factors associated with RDW, patients were subdivided into quartile groups according to the levels of RDW. Between the groups, we performed analysis of variance for continuous variables and Fisher’s exact test for categorical variables. The degree of leukoaraiosis is categorized according to its severity using the Fazekas scale (0–1 as mild degree, and 2–3 as severe degree). To evaluate the effect of RDW on leukoaraiosis, we conducted univariate and multivariate logistic regression analyses with the severity of leukoaraiosis (mild degree vs. severe degree) as a dependent variable. In the multivariate model, adjustments were performed for gender, age, and potential variables with p<0.10 in univariate analyses. To better understand the effect of RDW on leukoaraiosis, we illustrated a smoothing spline plot for the estimated probability for the presence of severe degree leukoaraiosis according to the RDW, based on the generalized additive regression model. The area under curve (AUC) was calculated as measure of discrimination by constructing the receiver operating characteristic (ROC) curve. Optimal cut-off value of RDW in the ROC curve was determined at the level with the highest Youden’s index (sensitivity+specifiity–1). We also constructed linear regression analyses treating the Fazekas scale (0–3) as a continuous dependent variable. The raw data for statistical analysis were presented in S1 Table. All statistical analyses were performed using the R package for Windows (version 3.1.2, R Foundation for Statistical Computing, Vienna, Austria). A two-sided p-value of <0.05 was considered statistically significant.

## Results

**Fig 1:**
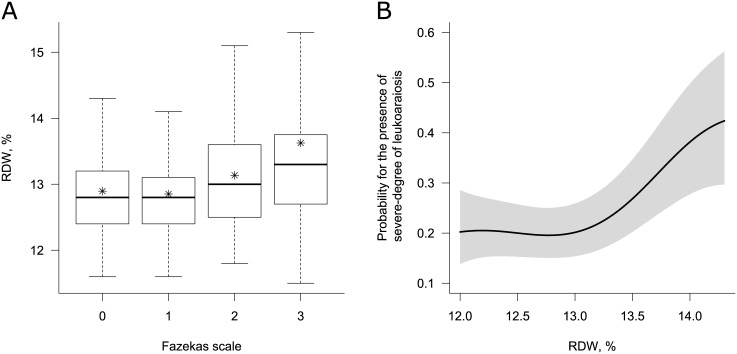
Relationship between red blood cell distribution width and leukoaraiosis. A) Boxplot for RDW according to the Fazekas scale. In each boxplot, the lower and upper ends of the box represent the 25^th^ and 75^th^ percentiles (interquartile range, IQR) and the line inside the box represents the median. An asterisk indicates the mean value. The ends of the vertical lines represent the lowest and the highest value within the range from 25^th^ percentile– 1.5 x IQR to 75^th^ percentile + 1.5 x IQR. B) Regression spline curve of estimated probability for severe degree of leukoaraiosis according to the level of RDW. The black lines and gray shadows represent the estimated probability and the 95% confidence intervals for the presence of severe degree of leukoaraiosis (Fazekas scale ≥2) at the RDW level, based on the generalized additive model with splines. Adjustments were made on the variables listed in Table 3. The x-axis is limited from the 5^th^ to the 95^th^ percentile of RDW to avoid overfitting by rare extremes. RDW indicates red blood cell distribution width.

A total of 1006 subjects were included in this study. Of these, 35.69% were male and the mean age was 64.34 ± 9.11 year. The mean value of RDW was 12.97 ± 0.86%. The prevalence of severe degree of leukoaraiosis (Fazekas scale ≥ 2) was 28.83%. Table 1 shows the clinical characteristics of the study subjects and differences by quartile groups of RDW. RDW was associated with old age, presence of DM, and severe degree of leukoaraiosis (p<0.05). Presence of SBI did not differ across the RDW quartiles (p = 0.712). The laboratory findings associated with RDW were glucose, MCV, MCH, and MCHC. To evaluate factors associated with severe degree of leukoaraiosis (Fazekas scale ≥ 2), logistic regression analyses was done. In the univariate analyses, severe degree of leukoaraiosis was associated with being female, old age, the presence of hypertension, cerebral artery atherosclerosis, SBI, high value of RDW and platelet count, and low value of hemoglobin and eGFR (Table 2). In the multivariate model with adjustments for variables with p<0.10 in the univariate analyses (gender, age, hypertension, DM, current smoking, cerebral artery atherosclerosis, SBI, hemoglobin, platelet count, glucose, and eGFR), RDW remained an independent risk factor for severe degree of leukoaraiosis (Table 3). Adjusted OR for 4^th^ quartile of RDW (> 13.3%) was 1.87 [95% confidence interval (CI), 1.20–2.92] with reference group of 1^st^ quartile of RDW (< 12.5%). When we treated RDW as a continuous variable, adjusted OR for 1% increment in RDW was 1.49 [95% CI, 1.24–1.82]. There was no significant interaction (p>0.05) between RDW and hemoglobin level in the multivariate model. On visual inspection of the estimated probability for the presence of severe degree of leukoaraiosis according to the RDW level, we could find a positive association between them and a threshold effect in the relationship above 13% of RDW (Fig 1). Indeed, the optimal cut-off point for RDW was > 13.0% based on the ROC curve with logistic regression for the presence of severe degree of leukoaraiosis (sensitivity, specificity was 47.9%, 69.7% at the cut-off, respectively. AUC of RDW was 0.58.).

**Table 1:** Clinical characteristics of participants according to the red blood cell distribution width.

Clinical characteristics	ALL (N = 1006)	Q1 (RDW <12.5%, N = 253)	Q2 (RDW 12.5–12.8%, N = 265)	Q3 (RDW 12.9–13.3%, N = 262)	Q4 (RDW >13.3%, N = 226)	P-value
Gender (male)	359 (35.69)	84 (33.20)	97 (36.60)	95 (36.26)	83 (36.73)	0.821
Age, year	64.34±9.11	62.91±9.04	63.95±9.28	64.91±8.98	65.72±8.94	0.005
Hypertension	582 (57.85)	141 (55.73)	167 (63.02)	153 (58.40)	121 (53.54)	0.162
Diabetes mellitus	232 (23.06)	76 (30.04)	59 (22.26)	53 (20.23)	44 (19.47)	0.023
Hypercholesterolemia	302 (30.02)	76 (30.04)	75 (28.30)	86 (32.82)	65 (28.76)	0.680
Current smoking	211 (20.97)	51 (20.16)	58 (21.89)	53 (20.23)	49 (21.68)	0.941
Coronary artery disease	49 (4.87)	12 (4.74)	9 (3.40)	10 (3.82)	18 (7.96)	0.112
Cerebral artery atherosclerosis	187 (18.59)	41 (16.21)	49 (18.49)	49 (18.70)	48 (21.24)	0.573
Silent brain infarction	234 (23.26)	54 (21.34)	63 (23.77)	59 (22.52)	58 (25.66)	0.714
Fazekas scale						<0.001
0	384 (38.17)	98 (38.74)	97 (36.60)	117 (44.66)	72 (31.86)	
1	332 (33.00)	90 (35.57)	106 (40.00)	77 (29.39)	59 (26.11)	
2	247 (24.55)	56 (22.13)	57 (21.51)	58 (22.14)	76 (33.63)	
3	43 (4.27)	9 (3.56)	5 (1.89)	10 (3.82)	19 (8.41)	
White blood cell count, x 10^9^/l	6.60±1.93	6.56±1.96	6.51±1.96	6.68±1.84	6.68±1.96	0.686
Haemoglobin, g/dl	13.49±1.41	13.56±1.27	13.52±1.38	13.57±1.45	13.28±1.52	0.094
Mean corpuscular volume, fl	91.60±4.13	91.61±3.50	91.92±4.15	91.92±3.99	90.86±4.78	0.016
Mean corpuscular hemoglobin, pg/cell	30.89±1.53	31.20±1.25	31.08±1.34	30.92±1.42	30.26±1.92	<0.001
Mean corpuscular hemoglobin concentration, g/dl	33.71±0.95	34.06±0.88	33.78±0.91	33.65±0.88	33.30±0.99	<0.001
Platelet count, x 10^9^/l	232.49±59.29	232.27±54.83	231.03±51.07	230.71±58.01	236.51±73.13	0.697
Glucose, mmol/l	7.14±2.72	6.88±2.53	7.09±2.73	7.02±2.58	7.64±3.02	0.013
Total cholesterol, mmol/l	5.03±1.02	5.04±1.00	5.01±0.97	5.14±1.09	4.93±1.02	0.174
Triglyceride, mmol/l	1.69±1.05	1.77±1.06	1.65±0.89	1.76±1.22	1.58±1.01	0.146
eGFR, ml/min/1.73m^2^	74.11±17.26	75.57±16.57	75.37±17.50	73.05±16.67	72.23±18.23	0.075

Values are number (%), or mean ± standard deviation. P-value is derived from Fisher's exact test or analysis of variance across the quartile group of RDW. eGFR, estimated glomerular filtration rate; RDW, red blood cell distribution width.

**Table 2:** Results of univariate logistic regression for the severity of leukoaraiosis.

Variables	Fazekas scale, 0 to 1 (N = 716)	Fazekas scale, 2 to 3 (N = 290)	OR [95% CI]	P-value
Gender (male)	272 (37.99)	87 (30.00)	0.70[0.52;0.94]	0.016
Age, year	62.15±8.65	69.73±7.91	1.11[1.09–1.13]	<0.001
Hypertension	389 (54.33)	193 (66.55)	1.67[1.26–2.23]	<0.001
Diabetes mellitus	155 (21.65)	77 (26.55)	1.31[0.95–1.79]	0.098
Hypercholesterolemia	211 (29.47)	91 (31.38)	1.09[0.81;1.47]	0.549
Current smoking	161 (22.49)	50 (17.24)	0.72[0.50–1.02]	0.062
Coronary artery disease	35 (4.89)	14 (4.83)	0.99[0.51–1.84]	0.984
Cerebral artery atherosclerosis	116 (16.20)	71 (24.48)	1.68[1.20–2.34]	0.003
Silent brain infarct	115 (16.06)	119 (41.03)	3.63[2.67–4.95]	<0.001
White blood cell count, x 10^9^/l	6.60±1.96	6.61±1.85	1.00[0.93–1.08]	0.948
Haemoglobin, g/dl	13.58±1.41	13.25±1.37	0.84[0.76–0.93]	0.001
Mean corpuscular volume, fl	91.55±4.04	91.74±4.33	1.01[0.98–1.05]	0.495
Mean corpuscular hemoglobin, pg/cell	30.90±1.48	30.86±1.64	0.99[0.90–1.08]	0.745
Mean corpuscular hemoglobin concentration, g/dl	33.74±0.93	33.64±0.99	0.90[0.78–1.04]	0.146
Platelet count, x 10^9^/l	228.85±54.66	241.48±68.70	1.00[1.00–1.01]	0.003
Glucose, mmol/l	7.24±2.79	6.91±2.54	0.95[0.91;1.01]	0.088
Total cholesterol, mmol/l	5.03±0.97	5.05±1.14	1.02[0.89;1.17]	0.756
Triglyceride, mmol/l	1.67±1.03	1.75±1.10	1.08[0.95;1.22]	0.241
eGFR, ml/min/1.73m^2^	75.98±17.01	69.50±17.05	0.98[0.97–0.99]	<0.001
RDW, %	12.88±0.70	13.21±1.12	1.56[1.32–1.84]	<0.001
RDW, quartile group				
Q1; <12.5%	188 (26.26)	65 (22.41)	Ref.	-
Q2; 12.5–12.8%	203 (28.35)	62 (21.38)	0.88[0.59–1.32]	0.546
Q3; 12.9–13.3%	194 (27.09)	68 (23.45)	1.01[0.68–1.51]	0.946
Q4; > 13.3%	131 (18.30)	95 (32.76)	2.09[1.42–3.09]	<0.001

Values are number (%), or mean ± standard deviation. OR and P-value are derived from logistic regression. CI, confidence interval; eGFR, estimated glomerular filtration rate; OR, odds ratio; RDW, red blood cell distribution width.

**Table 3:** Red blood cell distribution on the presence of severe degree of leukoaraiosis.

RDW	adjusted OR [95% CI]*	P-value
*As categorical variable*		
RDW quartile group		
Q1; < 12.5%	Ref.	-
Q2; 12.5–12.8%	0.76 [0.48–1.19]	0.231
Q3; 12.9–13.3%	0.83 [0.53–1.30]	0.412
Q4; > 13.3%	1.87 [1.20–2.92]	0.006
By optimal cut-off derived from ROC curve		
RDW ≤ 13.0%	Ref.	-
RDW > 13.0%	1.89 [1.37–2.60]	<0.001
*As continuous variable*		
RDW, %	1.49 [1.24–1.82]	<0.001

Values for RDW are derived from the multivariate logistic regression models which have the presence of severe degree of white matter disease as a dependent variable.

* adjusted for gender, age, hypertension, diabetes mellitus, current smoking, cerebral artery atherosclerosis, silent brain infarct, hemoglobin, platelet count, glucose, and eGFR. CI, confidence interval; eGFR, estimated glomerular filtration rate; OR, odds ratio; RDW, red blood cell distribution width.

As sensitivity analyses, we performed additional adjustments for MCV, MCH, and MCHC which might be potential confounding factors as hematologic markers of anemia. None of them changed significant effect of RDW in the sensitivity analyses (data not shown). In subgroup analyses according to gender and hemoglobin level, RDW was consistently associated with the presence of severe degree of leukoaraiosis (Table 4). However, the significance of RDW disappeared in subgroups with the lower half of RDW (≤ 13.0%), which implied the prognostic information of RDW was present only in those with an elevated value and threshold effect of RDW on leukoaraiosis. We constructed linear regression models which had the Fazekas scale (0–3) as a dependent variable, assuming that the scale was a continuous variable (S2 Table). In the univariate and multivariate linear regression analyses, we also found a significant positive association between RDW and the severity of leukoaraiosis.

**Table 4:** Subgroup analysis for the effect of RDW on severe-degree of leukoaraiosis.

Subgroup	N	RDW, %	
		OR [95% CI]	P-value
Gender			
male	359	1.82 [1.37;2.51]	<0.001
female	647	1.45 [1.19;1.80]	<0.001
Haemoglobin*			
> 13.4 g/dl	495	1.71 [1.28;2.30]	<0.001
≤ 13.4 g/dl	511	1.46 [1.20;1.81]	<0.001
RDW*			
> 12.8%	518	1.74 [0.01;2.28]	<0.001
≤ 12.8%	488	1.03 [0.51;2.07]	0.944

Values are derived from logistic regression models which have the presence of severe degree of leukoaraiosis as a dependent variable. OR is per one % increment in RDW.

*Cut-off is median value of hemoglobin and RDW. CI, confidence interval; OR, odds ratio; RDW, red blood cell distribution width.

## Discussion

We demonstrated a significant association between high RDW level and the severity of leukoaraiosis. The association remained significant even after allowing for multiple potential confounding factors, including the hemoglobin level, which suggested RDW might be an independent predictor for the severe degree of leukoaraiosis. As leukoaraiosis is considered to indicate reduced brain reserve capacity following injury, our finding could provide one explanation for the worse clinical outcomes in stroke patients with a high RDW [6,11,13]. To our knowledge, the association between elevated RDW level and severity of leukoaraiosis has not been reported before.

The strong prognostic value of RDW has been well established in the general population and in those with various medical conditions, especially vascular diseases [2–4]. Although the exact pathophysiological mechanism linking elevated RDW and severity of leukoaraiosis is still unclear, several hypothetical explanations have been suggested. One of suggested explanations is that RDW reflects an underlying inflammation [5]. Inflammation induces ineffective erythropoiesis and enables immature RBCs to enter the circulation, which leads to anisocytosis [16]. It is well-established that RDW is positively correlated with various inflammatory markers [23]. Enhanced inflammation is strongly associated with the presence or severity of leukoaraiosis, and those with inflammation are at increased risk for further progression of leukoaraiosis in the follow-up studies [24,25]. Recent studies have suggested that elevated RDW might also reflect impairment of microcirculation [26]. Over their life, RBCs gradually lose cell membrane deformability with age and eventually rupture and are eliminated in the spleen. When RBCs reach a small vessel, they must squeeze their morphology through the small diameter of the vessel and return to their original shape upon exiting it (RBC deformability) [27]. However, too stiff and fragile RBCs do not squeeze through the capillaries, and can impair blood flow through the microcirculation or block the small vessels [28]. Therefore, the RBC deformability plays crucially important roles in RBC passage and oxygen transport through the microcirculation. Elevated RDW is strongly associated with reduced RBC deformability [26]. As chronic ischemic disease and inadequate blood supply in the cerebral microcirculation are important mechanisms for the development and progression of leukoaraiosis, it is plausible that reduced RBC deformability impairs the cerebral microcirculation and subsequently leads to the development of leukoaraiosis in people with elevated RDW level.

There are many other medical conditions associated with RDW including aging, hematologic disease, liver disease, kidney disease, metabolic syndrome, arterial stiffness, infection, and impaired heart function, etc. [2,23]. These underlying factors might contribute to the association between RDW and leukoaraiosis. Our cohort had no serious medicosurgical disease at baseline examination, and RDW was independently associated with leukoaraiosis after adjusting for age, sex, cardiovascular risk factors, and eGFR. Anemia is considered most strong confounder for the prognostic value of RDW. In our subgroup analyses, RDW was significant independently to the hemoglobin level. Additional adjustments for MCV, MCH or MCHC, hematologic indices for anemia evaluation, also did not change the statistical significance of RDW. Even with the possibility of a residual confounding effect, the prognostic value of RDW seems to be independent of anemia, consistent with previous reports [2,29].

We should acknowledge several limitations in our study. First, a retrospective design means that a selection bias can be present. Furthermore, our study subjects were visitors to an out-patient department in a single centre; therefore their demographic characteristics were not identical with those of general healthy population. Also, our study subjects had a higher prevalence of cardiovascular risk factors than would be found in a general healthy population. A prospective observation conducted on the general population is required to validate our results in further study. Second, we did not measure the volume of leukoaraiosis using a computational quantitative method. A computational quantitative method might be more appropriate than a visual rating method to determine the severity and progression of leukoaraiosis. However, a quantitative method is laborious and difficult to perform in the clinical setting. Conversely, visual rating method has been widely used, and is a straightforward method for the assessment of the severity of leukoaraiosis. In our study, the inter-rater agreement was adequate and was comparable to those of the previous studies [30]. It is unlikely that the use of a visual rating method weakened our results since the visual rating method has shown a high correlation with quantitative method [31]. Third, although we collected and adjusted multiple risk factors and laboratory findings where possible, no data was available for endothelial function, thrombogenicity and other confounding factors. Vitamin B12 or folate may contribute to the significant association between RDW and leukoaraiosis [32]. In addition, lack of data regarding obesity would impact our result, because obesity or increased body mass index increases the risk of small vessel disease including leukoaraiosis [33]. Finally, we could not conclude whether RDW has a casual role in the development and progression of leukoaraiosis or is merely a simple parameter reflecting the degree of leukoaraiosis. To evaluate the exact mechanisms and casual effects of RDW on leukoaraiosis, there is a need for a long-term follow-up study including repeated imaging.

## Conclusions

In the present study, high RDW was independently associated with advanced leukoaraiosis in Korean population. Although further studies are needed to determine whether elevated RDW is clinically helpful to identify leukoaraiosis, elevated RDW may be one of the surrogate markers for advanced leukoaraiosis in neurologically healthy population.

## Supporting Information

S1 Table File containing patient information. (XLS)

S2 Table Result of linear regression model with Fazekas scale as a continuous dependent variable. Values are derived from linear regression models which have Fazeka’s scale (0 to 3) as a dependent variable. CI, confidence interval; eGFR, estimated glomerular filtration rate; RDW, red blood cell distribution width.
(DOC)
