# Supplementary material for: Red Blood Cell Distribution Width Is Associated with Severity of Leukoaraiosis
Source: PLoS One. 2016 Feb 26;11(2):e0150308. doi: 10.1371/journal.pone.0150308 (PMC4769290; doi:10.1371/journal.pone.0150308)
Supplement: S2 Table — Values are derived from linear regression models which have Fazeka’s scale (0 to 3) as a dependent variable. CI, confidence interval; eGFR, estimated glomerular filtration rate; RDW, red blood cell distribution width. (DOC) [file pone.0150308.s002.doc]

**S1 Table.** Result of linear regression model with Fazekas scale as a continuous dependent variable

| Variables | Univariate model | | Multivariate model | |
| --- | --- | --- | --- | --- |
|  | coefficient [95% CI] | P-value | coefficient [95% CI] | P-value |
| Gender (male) | -0.138 [-0.253;-0.023] | 0.019 | -0.077 [-0.197;0.043] | 0.207 |
| Age, year | 0.042 [0.037;0.048] | <0.001 | 0.035 [0.029;0.041] | <0.001 |
| Hypertension | 0.263 [0.152;0.374] | <0.001 | 0.032 [-0.070;0.134] | 0.541 |
| Diabetes mellitus | 0.116 [-0.015;0.247] | 0.082 | -0.020 [-0.14;0.098] | 0.744 |
| Hypercholesterolemia | 0.068 [-0.053;0.188] | 0.270 |  |  |
| Current smoking | -0.104 [-0.239;0.032] | 0.133 |  |  |
| Coronary artery disease | -0.054 [-0.311;0.203] | 0.680 |  |  |
| Cerebral artery atherosclerosis | 0.226 [0.085;0.368] | 0.002 | 0.012 [-0.115;0.139] | 0.856 |
| Silent brain infarct | 0.590 [0.464;0.715] | <0.001 | 0.448 [0.331;0.565] | <0.001 |
| White blood cell count, x 10^9^/l | 0.006 [-0.022;0.035] | 0.663 |  |  |
| Haemoglobin, g/dL | -0.073 [-0.112;-0.034] | <0.001 | 0.006 [-0.036;0.048] | 0.771 |
| Mean corpuscular volume, fl | 0.005 [-0.009;0.018] | 0.487 |  |  |
| Mean corpuscular haemoglobin, pg/cell | -0.010 [-0.047;0.026] | 0.570 |  |  |
| Mean corpuscular haemoglobin concentration, g/dl | -0.053 [-0.111;0.005] | 0.075 | 0.001 [-0.057;0.058] | 0.978 |
| Platelet count, x 10^9^/l | 0.002 [0.001;0.003] | 0.001 | 0.001 [0.001;0.002] | 0.001 |
| Glucose, mmol/l | -0.014 [-0.035;0.006] | 0.165 |  |  |
| Total cholesterol, mmol/l | 0.014 [-0.035;0.006] | 0.618 |  |  |
| Triglyceride, mmol/l | 0.003 [-0.040;0.068] | 0.900 |  |  |
| eGFR, ml/min/1.73m^2^ | -0.011 [-0.014;-0.008] | <0.001 | -0.003 [-0.006;-0.000] | 0.023 |
| RDW, % | 0.172 [0.108;0.235] | <0.001 | 0.108 [0.048;0.168] | <0.001 |

Values are derived from linear regression models which have Fazeka’s scale (0 to 3) as a dependent variable. CI, confidence interval; eGFR, estimated glomerular filtration rate; RDW, red blood cell distribution width.
